# Extracts and Gleanings

**Published:** 1873-09

**Authors:** 


### EXTRACTS AND GLEANINGS FROM OUR EXCHANGES.

### *MULTUM IN PARVO.*

Fischl on Pneumonia Migrans (*Prager Vierteljahrsschr.*, Bd. 114, S. 112–123).—Schiffer calls attention to this article, in a review in the *Central-Blatt fur Mediz. Wiss.*, No. 35, 1872. It appears that up to the present time only Waldenburg and Wiegand have contributed accounts of this rare affection; the former relating one case, and the latter two cases. The chief characteristic of the disease consists in many parts of the lung being successively attacked by the croupous process, the portions first attacked healing as the disease advances to the healthy parts.

The course of this disease may be anatomically continuous, as in the case of Waldenburg, where the disease began as a pneumonia dextra inferior, passed upwards through the entire right lung, attacked the apex of the left lung, and by steady advances reached the base of the lung on the left side. This course was passed over *twice* by the disease in the same individual. On the other hand, there may be no such regularity in the course pursued by the disease, and the same lung may be attacked first in one part and then in another. In any event, the disorder is very tedious, and, as Waldenburg has observed, highly suggestive of erysipelas migrans. The case related by Fischl, of a strong woman, thirty-six years old, showed the last-mentioned characteristics. When this patient was first seen, fourteen days after the beginning of the disease, the infiltration was discovered in the left lung, upper part; two days later, the infiltration was found at the base of the right lung; a few days subsequent to this, the disease was found in the infraclavicular region, left side, etc. Twice, parts were attacked which had been already affected. The infllirations were quickly developed, without initial chills, and were very quickly absorbed. The disease lasted six weeks, with continued fever, and the sputa continued of a catarrhal nature during the entire course of the affection, with at no time the appearance of any blood in them.

Treatment of Cystitis.—Dr. A. W. Rogers (*Medical and Surgical Journal*) says: In a pretty large practice of about thirty-five years' duration, I have met hardly any cases of acute cystitis, except such as supervened upon the prolonged application of a blister of cantharidies or the internal use of that or spirits of turpentine. I have met with many chronic cases and very many cases of dysuria, which are commonly regarded as cases of irritation of the neck of the bladder. But, no doubt many of the latter are true cases of a degree of cystitis. Many more of the latter occur in females than in males. Some of them have been accompanied with blood in the urine, which, from the attending symptoms I had reason to believe came from the bladder. All the cases I have had of an acute kind have been relieved, and the most of them got entirely well under the use of the following means: Rest, warm fomentations to hypergastric, pubic and perineal regions, and sometimes the moderately warm or tepid bath, warm demulcent drinks, small doses of spts. niter dulcis and tinc. opii. camph., equal parts, say ʒss. of each every hour; or pills of sulph. morph. gr. 1-20 to 1-10; or ext. hyoscyam. gr. $\frac{1}{2}$ to 1, one every two hours; or pills of camph. gr. $\frac{1}{2}$, ext. hyoscyam. gr. 1, one every two hours. The mixture of equal parts of spts. nit. dulc. and tinc. opii. camph. frequently given in small doses will relieve a large majority of the acute cases of dysuria or mild cystitis, for which the physician is called to prescribe, such as occur from cold, some temporary menstrual or sexual trouble, and will temporarily benefit all of them. Where this remedy does not answer, the morphia with camphor will often succeed.

For all bleedings from the urinary organs gallic acid has always proved a reliable remedy, one which usually acts very promptly. It has so often succeeded in my hands that I have not looked for another. But I have no doubt that ergot is also an efficient remedy and it is a matter of cougratulation that its use in all kinds of hemorrhages is being proved to be so effective.

The beneficial effects of ergot in paralysis of the bladder following retention of the urine I have proved in several cases. The judicious use of this remedy will often save the physician and the patient much inconvenience. By its aid the use of the catheter may much sooner be dispensed with.

Iodoform for Prurigo and Hemorrhoids.—An ointment made of iodoform (ʒi.) and simple cerate (℥i.) will be found useful in many cases of prurigo. The following is recommended when piles are very painful, especially during defecation: ℞. Iodoform, powdered, twenty grains; cocoa butter, ʒi. Melt and mix for suppositories.

THE INTERNAL USE OF ACONITE IN NEURALGIA.—I send you a few notes of cases showing the great use of tincture of aconite, both alone and in combination with other remedies, in neuralgia and muscular rheumatism. I now speak of its *internal* use, in contradistinction to *external.* I give five cases, each the representative of a different class of the same affection.

*Case* 1.—Mrs. D. had been for some time under my care for violent paroxysmal neuralgia of the face, partially due to carious teeth and partially to gastric derangement. As she would not have the bad teeth extracted nor tend to her digestion or bowels, I was perpetually trying to invent something new to mitigate the agony she suffered. On August 29th she began to take tinct. aconit., min. x.; tinct. colchici, min. iij.; brom. potass., gr. v., each third hour, with spt. chloroform min. xx. Having been prepared for this treatment by a purgative, on the 30th she had almost, and on the 31st completely, recovered; and this happy state of things lasted for six and weeks. Of course, in this case, extraction of the bad teeth proper attention to the digestive organs, would have afforded most hope of a radical cure. * * * * *

I have now given five cases, each a representative of a different class of rheumatic and neuralgic affections, in which I have found the internal use of tincture of aconite exceedingly useful. It remains for me to say in what class of cases I have not found such good effects. They are two: first, in acute rheumatism attacking the joints; and secondly, in lumbago, its internal use has proved of much less value. In the latter affection, however, complete relief may often be obtained by the application of chloroform liniment, aconit., and liniment. belladonnæ in equal parts on lint with oiled silk over it. In conclusion, I would add, that constipation, or a disordered state of the stomach, or both, should be remedied before commencing the use of aconite, or *at least* in such cases suitable remedies for the relief of such a state of things should be combined with it. As a rule, aconite seems to have more effect when combined with spirit of chloroform than when taken alone—at least, so far as my experience goes. It is well always to commence with a small dose, as I know of no remedy so variable in its effects on different constitutions. I have seen a single dose of eight minims produce decided tingling *all over the body* in an adult, and I have given as much as twenty minims each third or fourth hour, without any tingling at all.—*A. Leslie Means, M.D., in Med. Times and Gazette.*

DIABETES.—Dr. O. Shultzer claims great success in the treatment of diabetes under the free use of glycerin internally, with citric acid and abstinence from starchy food.

HEADACHE IN CHILDREN.—After remarking on the symptomatic value of "Headache" as a term, the author reviewed the commonest varieties of headache in children, and then dwelt especially on a peculiar functional form, of which he had met with several examples. These were associated, he believed, with some intricate, though undemonstrable, change, physical, structural, or chemical, going on within the cranium; giving rise to some confusion or abnormal sensation in the head or to actual headache. This peculiar cerebal change is accompanied occasionally, but not of necessity, with enlargement of the head, wasting of the extremities, flabbiness of the muscles, and looseness of the joints; there are also pallor and debility, restlessness by night, irritability by day; a slow and sometimes irregular pulse, with dull and persistent heache; the temperature is normal. Dr. Day treated the early stage of the disease with bromide and iodide of potassium, and advocated, later on the use of iron, bark, and cod oil. Tonics did harm in the early treatment.

Dr. Farquharson gave some interesting experience in relation to headache in children based upon cases treated by him at Rugby. He referred to the importance of not overlooking headache as a symptom of slight sunstroke, and in connexion with overwork; in the latter cases the urine contained alkaline phosphates.

Dr. Broadbent regarded persistent headache in a child as a suspicious symptom. Dr. Day's cases he considered examples of the headache, of rickets, or hydrocephalus. He advocated the use of small doses of mercury, continued for a long time.

TREATMENT OF FISSURE OF THE ANUS (*Medical Times and Gazette*, June 8, 1872).—A lecture on this subject, by Dr. Dolbeau, is reported by Dr. Osborne Powell. He observes that each evacuation of the bowels is accompanied by a feeling of something cracking, followed by intense pain; nevertheless, in many instances no fissure can be found, even after the most careful and minute inspection. He consequently regards it as of the nature of neuralgia. It commonly occurs between the ages of eighteen and thirty, and most frequently in women. In the treatment, the first thing to do is to abolish the muscular element. Récamier, whose name is inseparable from this history of this malady, well comprehended that the fissural lesion is an element without importance, and he cured several patients by what he termed *cadenced massage*. Chloroform was then unknown. He had the patient held by some strong assistants, and he introduced first one finger, then another, and so on, until he had passed his whole hand into the rectum; he then closed his fist, and drew his hand from the intestine. This was a most rational operation, and the employment of chloroform in the present day permits us to practice it in a much less barbarous manner. Thus, the patient being put under the influence of chloroform, the surgeon introduces his two thumbs into the rectum, and endeavors to bring them in contact with the two ischia; at that moment one hears a strong cracking noise, and the operation is finished. The first stool after the operation is painful, but this pain is simply the result of the region being contused, and does not in the least resemble the pain which characterized the spasmodic attacks. The succeeding stools are without any pain, and the cure is assured.

Cases of Traumatic Malignant Onychia Successfully Treated by Means of Nitrate of Lead.—In the *Ippocratico* Dr. D. Peruzzi has recorded a remarkable instance of the above, in the form of a letter addressed to Professor Vanzetti, who was the first in Italy to advocate the use of nitrate of lead in malignant onychia, and had been eminently successful in fourteen cases of the disease. Dr. Peruzzi's case was a very bad one; so bad, indeed, that amputation of the diseased part seemed the only possible procedure. It occurred in a youth of seventeen, of lymphatic constitution, who fourteen months previously had wounded the great toe of his right foot. The wound gradually extended and suppurated loosening the nail to the root, and producing hemorrhage, which became more and more frequent and abundant. The pain was most intense; sleep quite impossible; and the patient was finally reduced to a worn-out condition. All previous medicinial applications had failed. On May 14th the loose extremity of the nail was cut off, and nitrate of lead sprinkled freely all over the diseased parts. A bit of rag was simply applied. Strong pain was felt, but speedily abated. A black polished scar was produced, and fell off at the end of six days. The parts healed beautifully. A second slight application of the substance was made with the object of hastening and ensuing a cure. Dr. Peruzzi's case is followed by the relation of three other instances of cure recorded by Drs. Verardini and Casati.—*Lancet.*

Treatment of Chronic Nasal Catarrh.—Dr. Whittaker, (*Clinic*, July 12, 1873), advocates the treatment of certain cases of nasal catarrh by pressure. He does it for the same reason that the surgeon uses pressure to remove stricture of the urethra. The constriction is usually found in the inferior meatus. Bougies of different sizes and shapes are employed as the particular case seems to indicate. His success in the five cases thus treated has been very gratifying. The suggestion is surely worth a trial, as by present methods these cases are treated with most unsatisfactory results. *Detroit Review of Medicine.*

Bromide of Potassium.—Professor See, in one of his last lectures at the School of Medicine, expatiated upon the medicinal properties of the bromide of potassium—a drug so much in vogue, and yet so little understood. He said it was often prescribed in a most reckless manner, and administered with substances not only chemically incompatible with it, but whose therapeutic action is diametrically opposed to it. For instance, it is frequently ordered with, or as a substitue for, the iodide of potassium, and *vice versa*, and there is a prescription in Bouchardat's "Formulaire" which bears the name of a celebrated physician, containing these two salts in combination with the chloride of sodium and butter, which was to be eaten with bread as a substitute for cod-liver oil. The bromide of potassium acts specially as a sedative on the vascular and nervous systems, whereas the iodide is purely an alterative, operating on the general processes of nutrition and elimination. The bromide of potassium is essentially a vascular remedy. It is probable that through the nerves it acts on the muscular coats of the vessels, causing permanent contraction of the latter and consequent anæmia of the organs, a condition opposed to that produced by belladonna. It acts indirectly as a sedative on the heart, which it may stop, but only when given in enormous doses. As a soporific or narcotic, it is preferable to opium, particularly in children, as it does not produce headache or the other inconveniences of the latter. Its double action, as vascular and as a nervous hyposthenisant, renders it a most invaluable agent in all cases of neurosis accompanied with congestion of the nervous centers.—*Medical Times and Gazette.*

Electro-Cautery for Piles, etc.—Our *confrere*, M. Lucas-Championniere, reports in his *Practical Journal* that M. Verneuil, at the Lariboisiere, has treated piles by running through them at several points the electric-cautery, at a dull red heat, which obliterates the vessels without setting up active inflammation beyond. Atrophy follows. Encouraged by the constant and rapid success of this method, M. Verneuil has also applied it to erectile tumors. Some very important successes resulted, and only one accident. An infant at the breast, with an enormous rapidly growing erectile tumor of the cheek, was subjected to this treatment, after other means had failed; but unfortunately erysipelas came on, and proved fatal. The method is to cauterise the tumor in as many points as the surface admits, wait the falling off of the scabs, and then cauterise afresh wherever any vessels remain. Ordinary cautery may be used by mounted needles, but the electric instrument is far superior. It is heated instantaneously, and does not affect the surrounding tissues.—*Medical News.*

On the Choice of Anæsthmetics.—Our one reason for abandoning chloroform, is its danger. If we could get rid of this, it would certainly stand without rival. My own experience of chloroform has been very considerable, and for a great variety of cases. I have had but a single death in my own practice, but I have been the witness of several in the practice of others. I have also had among my own patients several very alarming occurrences, in which it was with the greatest difficulty that death was prevented. In the case of a girl at Reigate, in whom apparent death occurred during excision of the knee, we had to perform artificial respiration for at least ten minutes before any signs of reanimation occurred. In another case at the London Hospital, in an amputation of the thigh, the period during which I thought the patient dead, was almost as long. In both these instances, death-like pallor of face and absence of pulse (cardiac syncope) were the alarming symptoms; and in both, artificial respiration, irritation of the surface, and the use of brandy enemeta, were the means resorted to. In addition to them, I have had many cases in which the pulse failed temporarily, and in which we had to begin artificial respiration in order to ward off the more dangerous condition. During the last four or five years, I think I have had fewer alarming cases of this kind than formerly; and I attribute this, in part, to the skill and care of those who have given chloroform to me, and in part to the fact that I have been very particular as to the administration of stimulants (brandy) before using the anæsthetic. I have never, of late, used chloroform without knowing that my patient had fasted for some hours, and had taken a dose of brandy within the last half-hour. These seem to me the two principal points; but if you insist upon the one, (the fasting,) it is additionally important that you attend to the other. In a fasting and exhausted state of the system, we have the condition in which cardiac syncope is the most likely to occur.—*Clinical Lecture by Jonathan Hutchinson, F. R. C., in British Med. Journal.*

Liquor Picis Alkalinus.—The following formula was suggested by Dr. H. D. Bulkley, of New York, for preparing a lotion to be used in place of a French proprietary tar prepaaation of some repute: Take of tar, two drachms; caustic potassa, one drachm; water, five fluid drachms; mix and dissolve, for external use. This lotion mixes easily with water, and only moderately discolors the skin. After it dries, there remains very little of the tarry stickiness. Dr. B. claims that the caustic alkali united to the tar is more valuable than the latter alone as an anti-pruritic. The solution has been used with advantage in eczema, in chronic as well as in more acute forms. He considers it the best form for the external use of tar.—*Lancet and Observer.*

Muriate of Ammonia.—Dr. W. J. Barkas, referring to Sir Thomas Watson's recommendation of this drug in that variety of neuralgia effecting the teeth and alveola processes, says: The prescription I invariably give, is: ℞.—Ammon chlorid., gr. xx.; sp. chloroform, min. xv.; tinct. lavand. co., min. x.; aquæ, ad. ℥j. M. ter die sum. The chloric ether is added to render the mixture more palatable, and also on account of its action as a diffusable stimulant; for persons suffering from this complaint are often in need of such a fillip. From two to four doses of the above mixture are generally sufficient to effect a cure when all other treatment has totally failed. It is advisable that the remedy be not continued beyond six doses, not because there is any danger in its administration, but because I have found that if recovery has not then taken place, the drug is of no further benefit.

"When the pain is very acute, five minims of the liquor morphiæ hydrochloratis B. P., added to each dose, is of great service in its relief. The cessation of pain here is not due to the morphia, for such a minute dose, given alone, would not have the slightest effect, but it appears to quicken the action of the chloride.

"Upon observing the rapid recovery in the above class of cases, I determined to employ the chloride in the treatment of other varieties of neuralgia. The only varieties, however, that seemed to be much benefitted, were those in which there could be traced a history of rheumatism or gout, either hereditary or acquired. Of these forms of neuralgia, sciatica and lumbiago are the most frequent."

Treatment of Psoriasis.—Psoriasis is not in itself a serious disease says M. A. de Montméja, but it is obstinate, and those who have once been affected by it are very liable to relapses. It is often hereditary, manifesting itself only when the adult period is reached, after which it may be either intermittent or inveterate. In the present state of our therapeutical knowledge we must not imagine that we can effect a radical cure of psoriasis; we may clear the skin, or hasten the evolution of an attack, but it is impossible to prevent relapses. The treatment is divisible into local and general means. The general treatment consists in the administration of mild and frequently repeated aperients and of arsenical and sulphuretted preparations, as well as of those containing cantharides. M. Hardy prefers small doses of the arseniate of soda to the other preparations of arsenic. M. de Montméja has obtained considerable success from the employment of two drops of tincture of cantharides in a glass eau sucrée, the dose being increased up to thirty drops per diem. Its use, however, requires extreme care and vigilance. Copaiva is sometimes very useful when given internally.

Nitrite of Amyl as an Antispasmodic.—Leartus Connor, M.D., Professor of Physiology in the Detroit Medical College, (*Detroit Review of Medicine, Medical Record*), "extols this remedy in neuralgia of the heart, and mentions a case, accompanied by alternating attacks of asthma, in which prompt relief was afforded after the patient had inhaled from six to ten drops. In this history the subjoined points are of interest: Though no hereditary cause for angina could be traced, the life of the patient had been such as to most thoroughly exhaust the nervous system. There was no evidence of any heart-disease or ossification of the arteries. The attack came on after a course of intermittent fever, unchecked for two weeks. As it was accompanied by alternating attacks of asthma, gastralgia and intercostal neuralgia, this would point to the conclusion that these were all due to the same cause—some disease of the nerves supplying the heart, solar plexus and the lungs. The advantages of this remedy over any other antispasmodic are its more rapid action, and its freedom from any unpleasant sequelæ. In his use of nitrite of amyl, he has never been able to obtain any other therapeutic effects than those following the relaxing of spasm.

"Wm. F. Jenks, M.D., Philadelphia (*Philadelphia Med. Times*), narrates a case of puerperal eclampsia which was immediately and satisfactorily overcome by the inhalation of nitrite of amyl."—*The Medical Cosmos.*

A New Means of Relieving Toothache.—This remedy consists in the employment of injections introduced into the gum near the diseased tooth. Dr. Dop has tried these injections in about one hundred cases. In twenty cases he made use of morphia, which succeeded very well, but only for a time. Chloroform was far more successful, and is now exclusively used by Dr. Dop. It was eminently successful in sixty-two cases out of eighty. The injection is made with the small syringe commonly used in France for subcutaneous injections. Only two drops are put in at a time. The needle is introduced gradually, and must remain *in situ* a few seconds. On withdrawing it, pressure must be exerted on the gum with the finger. In by far the greater number of cases, one injection is quite enough to stop the toothache. In some instances a second injection is needed twenty-four after the first. During absence of pain, the tooth may be plugged, or dressed with creosote, etc.—*Revue Médicale de Toulouse.*

Wash for Failing Hair.—℞. Tincture cantharides, ℥iiss.; Jamaica rum (best), ℥ijss.; glycerin (best), ℥j.; sesquicarbonate of ammonia, ʒj.; oil origanum, gtt. xv.; oil rosemary, gtt. xv.; distilled water, ℥ix. M. S. Moisten, and rub the scalp two or three times a day.

Nurse's Sore-Mouth.—Prof. N. S. Davis (*Chicago Md. Ex.*) remarks: "Many years since, a careful study of the phenomena of the disease, led me to regard it as arising essentially from a deficiency of phosphatic salts in the blood. The woman, in furnishing from the blood the material necessary for the nutrition and growth of herself and child, does not assimilate these salts fast enough to supply the demand. The indication for treatment is chiefly to supply this deficiency; and no *tonics*, merely as such, which do not contain these salts as prominent constitnents, will do any permanent good. If, as soon as the patient begins to feel the scalding and tender sensations in the edges of the tongue and mouth, she is required to take, regularly, a fluid drachm of the compound syrup of the hypophosphites at each meal-sime, and at bed-time, with plain, nutritious food, and a little fresh, out-of-door air, every day, it will generally arrest the progress of the disease effectually, and she will remain well so long as she continues the medicine from two to four times a day. But in many instances it will be found necessary to keep up the supply of medicine during the whole period of nursing.

"In several instances under our care, the omission of the medicine for two weeks during any part of the time, would result in a renewal of the symptoms of trouble in the mouth and stomach; but with the renewal of the medicine the symptoms would disappear.

"There are several other prescriptions which will answer a similar purpose. One of the best, is the mixture of extract of malt, two parts, and compound syrup of hypophosphites, one part, given in doses of a small tablespoonful at each meal-time. If the disease is neglected, or treated with ordinary tonics, or stimulants, or both, until the mucous membrane of the mouth, fauces, stomach, etc., is already extensively ulcerated, and the blood of the patient extremely impoverished, no treatment may supercede the necessity for weaning the child.

Fluid Extrcat of Aconite as a Local Application.—For two years past I have made numerous experiments with aconite, topically applied. After requesting others to use it thus, I am enabled, from their experience and my own, to deduce the following results: All cases of swelled face arising from dental and neuralgic pains yield readily to a solution of the fluid extract and water, equal parts.—*Dr. E. J. Marsh, in Eclectic Medical Journal.*

Treatment of Pemphigus.—Mr. Hillairet (*Le Mouvement Medicnl,* September 14, 1872), reasoning from the analogy between this disease and burns of the second degree, believes that a similar treatment—dressing with cotton-batting (*ouate*) and lime-water liniment—will be found to answer well in both.

Electricity as a Means of Resuscitation.—Allan M'Lane Hamilton, M.D., New York (*American Practitioner*, October, 1872), in a paper on this subject, speaks of the following results arrived at from his own practice and experiments: 1. That it is useless to expect good results if five minutes have elapsed since life appears extinct. 2. That the current should be applied faithfully and steadily, one pole being placed on the ensiform cartilage, the other on the base of the skull or over the tracks of the great nerves of the neck. 3. That the faradic and interrupted galvanic currents are the best. 4. That the current should be applied some time after respiratory movements have become regular.

In concluding, the author says: The necessity of having a battery within reach is apparent. Every practitioner should have a small one for emergencies. They should be kept at each life-saving station on the coast, ready charged, with directions for immediate use. If this were done, he doubts if the percentage of deaths would be so great as it now is. Artificial respiration by the production of muscular movements is a very valuable means of restoration; but a force that acts directly upon the nerves supplying the muscles of respiration, is by far the surest and the best.

Tetanus.—M. Demarquay has communicated to the Medical Section of the Academy of Sciences two cases of traumatic tetanus successfully treated by intramuscular injections of morphine. He began by an injection, by means of the usual hypodermic syringe, into each masseter, and into the muscles of the neck on each side of the spinal column. The wound being painful, he also injected morphia into the muscles in its neighborhood. Immediate relief followed. When the contractions returned after a few hours, the injections were repeated, and whatever muscles suffered were thus treated. Thus, the muscles in the region of the back, the loins and the abdomen, were injected, as was also the sterno-clydo-mastoideus; while the course of the diaphragmatic and pneumogastric nerves were respectively selected for the purpose of restraining spasm of the diaphragm, and the difficulty of deglutition from the contraction of the œsophageal muscles.

Prurigo.—Dr. Rothmund, (*London Med. Record*) advises the internal administration of carbolic acid, which he says excels every other method of treatment for prurigo. He also advises the hypodermic injection of carbolic acid, which, he says, causes no irritation in the parts.

Painting the parts, after thoroughly drying them, with collodion, made elastic with castor oil, has proven successful in some obstinate cases. When the film comes off it should be reapplied, and all cracks in it repaired at once.

Gleet Treated with Medicated Bougies.—G. Lorey gives the results of eighty cases of gonorrhœa and gleet treated by this means. The cases of gleet, twenty in number, were all cured in a short time; the longest course included twenty-two bougies, one a day, and the shortest three bougies, the average being nine. The author observes that these cases, being treated in a hospital, doubtless derived benefit from the regular life there; it is not uncommon for a gleet to be greatly exacerbated by a long walk, slight excess in drinking, or a single act of coition. The bougies used were seven and a quarter inches long—i. e., about the length of the urethra—and from one-eigth to one-sixteenth of an inch in diameter. The center was of gelatine, the outside of gum-arabic mixed with the medicine, three-fourths of a grain, each, of sulphate of zinc and belladonna. After being dipped in cold water, they are easily inserted. In the sixty cases of gonorrhœa, no such startling results followed. The bougies served as well as the ordinary injections to cut short the disease—no better. But for two of the incidents of gonorrhœa, pain in making water and nocturnal erections, bougies medicated with opium (three-fourths grain), or opium and belladonna, (aa., three-fourths grain), acted admirably. Put in at night, they insure comfortable rest and easy micturition in the morning. It has been urged that, like permanent bougies, they might produce orchitis; but they are dissolved in the course of an hour and a half; and no orchitis occurred in any of the eighty cases observed by M. Lorey. *Annales de Dermatolugie et de Syphilographie.*

Danger of Performing Operations on the Cervix Uteri.—Dr. Alphonse Leteinturier has published a valuable monograph of the above subject, the conclusions of which may be thus summed up: All operations, even the slightest, bearing on the neck of the uterus, may be the starting-point of grave accidents; in such cases we generally find a more or less old affection of the uterine annexes. The operation performed on the cervix seems to stir up a former inflammatory process which had subsided. This latter fact may be explained by the existence of one of the three following conditions: lymphangitis, extending from the cervix uteri; general congestion of the pelvic organs; congestion localized in some points of the genital system, and determined by reflex action proceeding from the cervix.—*Lancet.*

Iron in Rheumatism.—Dr. J. R. Reynolds recommends the perchloride of iron in rheumatism, in thirty to forty-drop doses every six hours. He says it relieves the joint affection in one to three days, and the fever subsides in about five days. It should be given early.

TREATMENT OF VALVULAR DISEASES OF THE HEART.—In one of his lectures, Cantani gives some hygienic directions to patients affected with heart disease. Physical and moral *repose* are indispensable to such patients; they may, indeed, enjoy moderate exercise, but without fatiguing themselves; moral emotions ought carefully to be avoided, as well as muscular efforts. When the patient shall feel tired or have palpitation of the heart, he shall keep in bed for a longer or shorter time, which plan suffices sometimes to moderate the palpitations and strengthen the systole. The patient to live in a temperate country, and avoid equally cold and excessive heat, and give up tobacco smoking.

The bowels should be kept open, since materials accumulated in the intestines may prevent the circulation of the blood in the vena-portal system. The food taken by such patients should be easily digested and rich in albumen, to sustain the nutrition of the body; meat, eggs, and milk are useful, but very little bread, pastry, or flatulent vegetables should be used.

Patients to eat often and little at a time, so as not to permit of great distention of the stomach, which may push up the diaphragm. A most moderate quantity of all substances which excite the circulation must be used, such as wine, hot food or drinks, tea, coffee, etc.

Digitalis may be given to moderate the frequency of the pulse, united with wine, ammonia, or ether.

In cases of anasarca, diuretics must be used, and the extremities enveloped in warm sheets. If the diuretics fail to make the œdema disappear, and if it be considerable, acupuncture may be made use of to evacutae the serosity. We should have recourse to this method, especially in œndema of the scrotum and prepuce. If this be followed by erysipelas, lead lotion may be applied on compresses. *Doctor.*

REMEDY FOR INFLUENZA.—Dr. Allan McLane Hamilton, writing under date of February 17th to the *Medical Record*, recommends the following treatment for this annoying affection. His directions are: Prepare the following mixture—℞. Carbolic acid, gtts. x.; tr. iodine, chloroform, aa. ʒij. Put a few drops of this mixture in a test-tube, and heat them over a spirit-lamp; when volatilization occurs, the mouth of the test-tube should be held beneath the nostrils. This operation is to be repeated every two or three hours. A violent fit of sneezing occurs at first, which is afterwards succeeded by a diminution of the symptoms.

SWEET TINCTURE OF RHUBARB.—℞. Rhubarb, bruised, liquorice, bruised, each ℥ii.; aniseed, bruised, sugar, each ℥i.; dilute alcohol, O ii. Macerate fourteen days, express and filter.

SYPHILITIC SKIN DISEASE.—With regard to the employment of iodide of potassium in the treatment of syphilitic skin diseases, Dr. Anderson lays down the following rules:

1. The longer the interval which has elapsed between the contraction of the syphilitic taint and the development of the eruption, the more confidently may we substitute it for mercury.

2. If the patient is cachectic, it is, as a rule, to be preferred to mercury, except in recent cases of syphilis, when the mercurial vapor bath, or some such treatment, is more likely to prove successful.

3. The more extensive the tertiary eruption, the more certain it is to yield to iodide of potassium, although to this rule there are numerous exceptions.

4. If there is any tendency to syphilitic disease of the nostrils or neighboring parts, iodide of potassium should be withheld, or given with great caution; for if it produces coryza, it is very apt to aggravate the morbid condition of the parts.

5. It should be given in full doses. Dr. Anderson's experience has led him to conclude that ten grains is the proper dose in the majority of cases, and that occasionally as much as thirty or forty thrice daily may be requisite. It is generally advisable to prescribe it in combination with a bitter, and in cachectic subjects a little iron is a valuable addition, as in the following prescription: Ammonio-citrate of iron, three drachms; iodide of potassium, one ounce; syrup of ginger, six ounces; compound infusion of gentian, eight ounces; water to twenty-four ounces. A tablespoonful in a large wine-glass full of water thrice daily.

TONSILITIS.—In the Leavenworth *Medical Herald* (April, 1873), Dr. S. H. Roberts strongly recommends the use of turpentine externally in tonsilitis. He folds flannel to four thicknesses, wrings it out in hot water, and pours oil of turpentine over a spot the size of a silver dollar. The flannel is then applied over the sub-parotid region, and the fomentation continued as long as it can be borne. After removal, a dry flannel is applied, and the same region rubbed with turpentine every two hours. This application is continued daily till resolution occurs. The doctor believes, from the evidence of his long experience, that thus applied early in the disease, the oil of turpentine has almost a specific effect in tonsilitis. That its action is not simply that of an irritant, he has proved, by employing mustard, croton oil, tincture of iodine, etc., in the same class of cases. They always failed to diminish the inflammation of tonsils, while the turpentine succeeded.

CHRONIC RHEUMATISM.—℞. Compound syrup stillingia, ℥ iii.; vin. rad. colchicum, ʒ iss. M. S. Teaspoonful thrice daily, and alternate with carbonate of lithia.

Loco-motor Ataxia.—Dr. W. Lambert (*Canda Lancet*, May, 1873,) writing of a case of this disease under treatment, remarks: As soon as I recognized the disease, I gave potass-bromid. grs. xv., ter in die, and submitted the patient to the action of magneto-electricity, once every twenty-four hours. I also gave two pills of aloes and iron, which produced too much relaxation, the effect continuing two or three days. This, in fact, seemed to prostrate her to such an extent that she was obliged to take to her bed, and there remain for a time. Fortunately, just then I received the September number of the *New York Medical Journal*, and in it saw that Dr. Desjardin Baumetz had given phosphorus in this disease, with excellent effects. I immediately ordered acid-phosphoric-dil., m. xv., ter in die, in simple syrup. The next day her menses came on, and in a short time she began to improve. In a few days I increased the dose to twenty-five and then to thirty minims. After ten or twelve days, I omitted the acid, and gave her the pyro-phosphate of iron for a week, and then returned to the acid. I continued the electricity every alternate day. In two weeks she was able to sit up, and had sufficient control over the muscles of her upper extremities to be able to knit. In one month she could walk about the house tolerably well. Now (December, 1868) it is something over two months, she can take long walks, do housework almost as well as ever, and has become very fleshy. The electricity has been discontinued for about one month, and she is not at all regular with her medicine at the present time. However, I have the most sanguine hopes that she will perfectly recover. The improvement has been so great, that it is impossible to discern anything wrong with her, except a very slight irregularity in her walk. By the middle of January, 1869, she had perfectly recovered, and has remained so up to the present time (February 2, 1873). This was the first case on record, and the first time that phosphoric acid had been used for this malady.

Cotton-Wool Dressing.—M. Verneuil, of Lariboisière, generally makes use of the cotton-wool dressing whenever it may be conveniently applied, but for such regions as the face and neck, where the above plan is not applicable, he uses a simple dressing of gauze, covered with a piece of oil-silk, which from time to time (three or four times a day) is removed, whilst the following liquid ($\frac{1}{2}$ to $2\frac{1}{2}$ drachms) is thrown on the gauze by means of a pulverizer: Water, 1,000 grammes; alcohol, 200 grammes; carbolic acid, 2 to 1g grammes.—*Journal de Médecine et de Chirurgie Pratiques.*

Glycerole of Iodine is used for loss of voice. It is composed of a solution of sixteen grains of iodine in one ounce of inodorous glycerin.

Tracheotomy by the Gavano-Cautery.—*La France Mèdicale* mentions the operation of tracheotomy as successfully performed by the galvano-cautery on a boy, æt. 13, who had a little pebble in the trachea for upwards of a month. The operation was thus performed by M. Amussat: a strongly curved needle was introduced through the tracheal tissue, comprising part of the windpipe itself; this needle was furnished with a stout double thread of platinum. When the needle had been passed through, the two ends of the thread were joined by being seized in a forceps which was in connection with a galvanic pile, and the section was performed by the heated wire without the loss of any blood. The trachea was thus opened, and directly the foreign body was forcibly expelled by an excess of coughing. Since this occasion the operation has been performed frequently, viz., five times by M. Verneuil, once by Prof. Voltolini, once by M. E. Bourdou. The *modus faciendi*, however, of M. Verneuil is somewhat different—he divides the operation into three stages, and proceeds from without, inwards. 1st. He divides the skin and soft parts; 2nd. The trachea; the 3rd stage is the introduction of the canula, the precaution chiefly to be taken being that of not injuring the posterior wall of the trachea by too hastily or too deeply entering the tube itself with the galvano-cautery.—*Doctor.*

Thoracic Aneurism.—Dr. McCall Anderson, in the *Glasgow Medical Journal*, February, 1873, mentions a case of thoracic aneurism, in which electrolysis was had recourse to as a last resort. A Stohrer's battery was used, with only a single insulated needle connected with the positive pole. The point of insertion was previously frozen by means of Richardson's spray apparatus. A zinc plate, connected with the negative pole, was placed on the chest, about seven inches from the point of insertion of the needle, and separated from the walls of the chest by a sponge dipped in salt and water. This was repeated four times, and the result was the reduction of the tumor to one-fourth its former size. It became quite solid, and firmer than the surrounding structures, while the pulsation and systolic murmur became less distinct, the purring tremor entirely disappeared, and the patient was relieved of all pain and discomfort, and felt in perfect health. Dr. A. thought that, in carrying out the operation, the object should be to induce only a partial coagulation, in the hope that this might be followed by a slow deposition of fibrin in successive layers. Sudden coagulation would tend to produce inflammation and sloughing.

Hair Restorative.—Petroleum is said, by repeated applications, to restore the hair.

THE NATURE AND TREATMENT OF THE CONSTITUTIONAL FORMS OF ECZEMA.—Professor Mapother, of Dublin, remarks in the *Medical Press*, of the 19th ult., that the following facts seem to prove that the gout poison is the cause of eczema:

1. Many other reliable observers have obtained uric acid and urates from the exudation of eczema, and their increase in the urine in the chronic stage of each disease is undoubted.

2. There is great increase of fibrin in the blood, and it exudes and spontaneously coagulates on the raw surface.

3. Both diseases are characterized by great tendency to œdema and desquamation; which latter, of course, is universal in eczema, and occurs in three-fourths of the cases of gout when localized.

4. Gout can be shown to be hereditary in about three-fifths of the cases, and such predisposition can be shown in about an equal proportion of cases of general eczema. The greater proneness of the male sex is observable in both diseases.

5. Every one must have remarked the frequent concurrence of symptoms of gout or of rheumatic gout and eczema. The Chelsea pensioners and the poorer agricultural people of this country exhibit this concurrence on the lagest scale. I have seen very few cases of general eczema which had not been preceded or accompanied by what is so well known as acid or gouty dyspepsia.

6. It is an aphorism of Hippocrates that gouty attacks are most frequent in spring and autumn, and the same may be undoubtedly said of eczema.

7. The parts most distant from the circulatory force of the heart and least vascular—for example, the extremeties and ears—are the most frequent seats of each disease, as the urates are most easily deposited.

8. And lastly, the treatment proven to be useful in gout is usually successful in eczema.

Dr. Mapother adds:

Lithia I have found of greatest use, as would be anticipated from its extraordinary powers of combining, and dissolving with urate of soda and uric acid. It never fails to act as a diuretic, and the derivative influence from the skin to the more extensive surface of the kidney can be easily understood.

MEASLES.—Carbonate of ammonia is given in doses of two to ten grains in simple solution every three or four hours, and it is claimed that the usual sequelæ do not appear. It should be given alone. Acid drinks or fruits neutralize its effects.

OIL OF CLOVES is very effectual in protecting animals against flies and musquitoes. A few drops rubbed on the skin are sufficient for a whole day.

A Remedy for Colds.—Dr. Dobell, in his work on Winter Cough, declares that colds can be stopped without lying in bed, staying at home, or in any way interfering with business—provided the treatment be begun directly the first signs of catarrh show themselves in the nose, eyes, throat, or chest. When the cold has become established, it will not answer. The treatment is as follows:

1. Give five grains of sesquicarbonate of ammonia and five minims of liquor morphia in an ounce of almond emulsion every three hours.

2. At night give an ounce and a half of liquor ammonia acetatis in a tumbler of cold water, after the patient has got into bed and been covered up with several extra blankets; cold water to be drunk freely during the night should the patient be thirsty.

3. In the morning the extra blankets should be removed so as to allow the skin to cool down before getting up.

4. Let him get up as usual and take his usual diet, but continue the ammonia and morphia mixture every four hours.

5. At bedtime the second night give a colocynth pill. No more than twelve doses of the mixture from first to last need be taken, as a rule; but should the catarrh seem disposed to come back after leaving off the medicine for a day, another six doses may be taken and another pill. During the treatment the patient should live a little better than usual, and on leaving it off should take an extra glass of wine for a day or two.

Galvanism of the Sympathetic in Grave's (Basedow's) Disease.—Dr. M. Meyer (*Berliner Klinische Wochenschrift*) records four cases of Graves's disease in females, where brilliant results were obtained by galvanizing the cervical sympathetic for a considerable time (during 20, 72, 60 and 84 sittings respectively in the four cases). A weak current was passed through the two sympathetics; then the one pole was applied to the submaxillary region, and the other to the shut eye, or to the goitre on the corresponding side; and the current passed for from two to three minutes. The effect of this treatment upon the dispersion of the exophthalmos and goitre is described as excellent—often after but a few sittings. The influence was also great upon the general health and upon the menstrual derangements, but less upon the palpitation.

It is remarkable that in all four cases the right side was especially affected, and that in one case there was no goitre.

Morfit's Hair Dye.—Infuse black tea, two ounces, in one gallon of boiling water; strain, and add three ounces of glycerin, half an ounce of tincture of cantharides, and one quart of bay-rum. Digest this mixture for a couple of days, and perfume with essence of rose or bergamot.

Treatment of Varicose Ulcers.—Dr. C. C. F. Gay, *Buffalo Medical and Surgical Journal*, May, 73, writes: My own method is that recommended by Gross. Like this distinguished surgeon, I am able to say that all my cases have been successful, but must add, that two of them were not altogether devoid of danger. Unless the patient be cautioned against such contingency, there may arise a venous hemorrhage that might prove disastrous. It is well, therefore, always to instruct patients how to apply the finger or make compression over the orifice, in order to arrest bleeding, in case of such accident.

I use the potassa. cum calce, rubbed up with alcohol, and apply it over the veins through an opening the size of a small pea made in adhesive plaster, taking care not to get the eschars in too close proximity, since danger might arise from two or three eschars running into each other as their surfaces enlarged, thus obtaining so large an eschar that it would be as difficult to close as the original ulcer. I usually place the eschars three or four inches apart, certainly no nearer together than this—sometimes much further apart.

I think it unsafe to use more than five or six eschars lest erysipelas might supervene, and this number may be used simultaneously upon both legs. In twenty minutes the potash is washed off with vinegar, and the slippery-elm poultice applied over the whole leg, including eschars and ulcers. In from three to four weeks I have invariably found that the ulcers have kept pace with the eschars in the reparative process, and that both have closed; and in not a single instance yet have I seen any return of the ulcerative process.

Arsenic in Hydrophobia.—In a late number of the *Correspondenz Blatt* Dr. Guisan gives a number of cases showing the value of arsenic as a prophylactic in hydrophobia, and even as a remedy also after the symptoms are marked. He relates that a rabid dog, between the 7th and 9th of June, bit thirteen persons in various towns of the canton of Freiburg. All were recommended to be treated with one-twentieth of a grain of arsenic morning and evening, as a prophylactic measure. Eight submitted to this prophylactic measure, and none were affected. Four declined, or were not allowed to take the arsenic. Of those four, two remained unaffected, and two died. One began the arsenic treatment, but speedily left it off; she was attacked, but at a much later period, and died. Dr. Guisan recommends not only the internal employment of the arsenic, but that the wound should be dressed with it.

Gelseminum as an Antiperiodic.—Dr. William W. Murray, of Baltimore, in a recent article, (*Medical and Surgical Reporter*), reports five cases of malarial fever, three of intermittent, and two of remittent, which were rapidly and effectually cured by the use of gelseminum.

Variola and Typhus Fever.—In the *Berliner Klinische Wochenschrift*, Dr. Th. Simon states that an individual laboring under a recent slight attack of small-pox, was admitted as a patient into the Hamburg Hospital. Shortly after his reception there were developed in him the premonitory symptoms—intumescence of spleen, disturbance of sensorium, and the peculiar aspect of the stools—of fever, evidently of the typhus type. During the vesication of the pustules, there made its appearance a very copious eruption of roseola. The case terminated favorably.

Dr. Simon took opportunity to state that he had, during the prevalence of the variolous epidemic in Hamburg, frequently witnessed the early and wide-spread prevalence, among typhus patients, of a roseolar eruption, most probably the result of an influence exercised by the presence of the variolous epidemic.

Infusion of Wild Cherry.—Powdered wild cherry bark, No. 60, one-half troy ounce; glycerin, two fluid ounces; water (temperature 86°), water (cold), each sufficient quantity. Moisten the bark with six fluid drachms of water at temperature 86°. Allow the mixture to stand for two hours in an air-tight vessel at about the same temperature; then pack it firmly in a glass percolator, mix the glycerin with ten fluid ounces of water at the temperature of 86°, and gradually pour the mixture upon the bark; and when it has all passed from the surface, continue the percolation with water until one pint of infusion is obtained.

Cod-Liver Oil Jelly.—At a meeting of the Medical Society of Stockholm, Sweden, this article was exhibited, as prepared by E. Queru, of New York city, and the following was stated to be its composition: Cod-liver oil, eighty-five parts; isinglass, three parts; sugar, eight parts; water, four parts. As there exhibited, it was a semi-transparent jelly, of a yellowish-green color, having a strong odor, but less strong taste of the oil. The advantages claimed for it are, easy administration and complete assimilation, and its retention by the weakest stomach.—*Pharmacist.*

Churchill's Syrup of the Hypophosphites.—℞. Hypophosphite of lime, ℥ iss.; hypophosphite of soda, ℥ ss.; hypophosphite of potassa, ʒ ss.; hot water, ʒ xx.; sugar, ʒ xxx.; orange flower water, ℥ i. Mix. This may be used in all cases where the hypophosphites are indicated except, perhaps, in phthisis. In this disease the hypophosphite of potassa is injurious, and should be omitted from the formula (or the hypophosphite of soda increased ℥ ss.) when used for the purpose. The formula is a valuable one for many conditions.

CARBOLIC ACID IN BRONCHITIS.—Dr. J. A. Liddell writes to the New York *Medical Record* as follows:

"In a bad case of chronic bronchitis—a case in which there was strongly-marked bronchiectasis on both sides, harassing cough both day and night, profuse muco-purulent secretion that oftentimes was very offensive in smell, and emaciation with other very general signs of bronchial phthisis—the writer has recently administered carbolic acid by inhalation, and made the patient comfortable by so doing, when every other palliative had failed.

"At first it was given in the vapor of hot or warm water; but after a short trial these inhalations were discontinued, because they made the patient perspire too much. Then it was administered in the form of spray with Codman and Shurtleff's atomizing apparatus No. 5, and the result was gratifying in every respect. The preparation which was used mostly consisted of the crystalized acid dissolved in water in the ratio of one grain of the former to one ounce of the latter; that is, one part of the acid to 480 parts of water. Trials were also made with a solution as weak as one part to 600, on the one hand, and as strong as one part 300, on the other; but those having a strength of one part to 450 or 480 answered best. The patient was made to breathe or inhale the spray with deep inspirations, from five to ten minutes at a sitting, unless a feeling of drowsiness were sooner produced, once a day usually; twice a day, however, when the expectoration was very profuse or offensive in smell.

VERATRUM VIRIDE IN ACUTE RHEUMATISM.—Heuser (*Allg. Med. Centralzeit*, Jan. 1873) considers veratrum viride to be superior to any other remedy in acute rheumatism. He uses a mixture of 1 part of the tincture with 4 of rectified spirit and 5 of water. Three to five drops of this mixture are given every two hours, and the joints kept warmly rolled up. The author has also observed the best effects result from its use in pleurisy, and considers that it will supersede digitalis in pneumonia and bronchitis. He warns against the use of large doses, which impair the remedial action of the drug. Five drops of the tincture reduced the pulse rate to one-half, and produced vomiting, weakness, cold sweats, a feeling of tearing and numbness in the extremities, and muscæ volitantes.—*London Med. Record, March* 19, 1873.

DIARRHŒA MIXTURE.—The following is said to be "infallible": ℞. Terræ japonicæ, ℥ viij.; radicis rhei, ℥ ijss.; gummi opii, ℥ iss.; spt. camphoræ, O iss.; spt. anise, O iss.; spt. vini rect., O ijss.; aquæ, O ijss. Mix and filter. S. A teaspoonful (for adult) in sweetened water.

THE TEMPERATURE IN DIPHTHERIA (by Dr. G. Faralli: *Le Mouvement Medical*, April 26, 1873; from *L'Imparziale*, March 1, 1873).—The author studied an epidemic of diphtheria with a view of noting the state of the temperature—a subject which has given rise to much discussion. His observations are as follows:

1. At the onset, chills, vomiting, convulsions, delirium, and in a few hours the thermometer rises to 40° (C). From this moment the temperature gradually falls until the third or fourth day, when the patient becomes apyretic (beningnant form).

2. In other cases the temperature rises again on the fourth day, but never to the degree reached at the onset of the disease. This is due to the formation of new diphtheritic patches on the side previously unaffected, or to the engorgement of glands.

3. This secondary infection is particularly evident in the typhoid form, the column of mercury continuing to ascend until the moment of the patient's death.

These characters are subject to variations from complicrtions; as, for example, in cases where stenosis of the larynx causes death, the temperature may remain normal.

LOCAL EMPLOYMENT OF CHLORATE OF POTASH IN CANCEROUS SORES.—In the *Berl. Klin. Wockenschrift*, No. 6, 1873, Dr. Burow, of Königsberg, advocates the local employment of chlorate of potash in the treatment of cancerous sores. His proceeding consists in sprinkling the sore with chlorate of potash in powder or crystals, and covering the whole with a wet compress. As the crystals of chlorate of potash exert a more powerful action than the powder, and excite greater pain, Dr. Burow uses the powder first, and replaces it by the crystals when sensibility has been abated. One of the cases was a cancerous sore of the left arm, which healed completely after eight weeks' treatment. Three other cases were cancerous sores of the breast; one was lost sight of, the other two are under treatment, and healing well. The fifth case recorded was connected with a cancer which originated in the periosteum of the upper jaw and left cheekbone, and then became ulcerated; in this case healing was complete in three months.—*The Lancet, April* 12, 1873.

LIME JUICE AND GLYCERIN.—℞. Lime or lemon juice, half a pint. Heat in a porcelain mortar to near the boiling point, and gradually add: Rose water, elder-flower water, rectified spirits, each ℥ii. Agitate the whole together. After twenty-four hours' repose, decant or filter through calico or muslin; then add: Glycerin (pure), ℥iiss.; oil of lemon, ʒss. Again agitate them together for some time, and, by careful manipulation, a milky liquid results, but it should be quite free from coarse floating sediment.

Chloroformization during Sleep.—Dr. W. M. Whitmarsh states, (*Lancet*), " having occasion to perform circumcision on a very nervous child, aged six years, and the evening being selected by the parents for the operation, I found on my arrival the little patient fast asleep. Not wishing to lose so good an opportunity, I, with my friend Mr. Gandy, thought it advisable to administer chloroform at once. This was done by pouring ten drops on a piece of lint, and repeating it until one drachm had been given, when the patient was thoroughly under its influence. The operation was then performed, and the patient dressed, not waking till half an hour after. The pulse did not appear to differ from that ordinarily observed during the administration of chloroform. It would be interesting to know if this mode of giving chloroform has been noticed by the profession, and whether in nervous patients and young children it would not be preferable to the shock to the system occasioned by fright and fear of suffocation."

Local Treatment of Skin and Syphilitic Diseases.—In an article published by Dr. Gamberini in the *Giornale Italiano delle Malat. Ven.*, the author gives a list of the various cases treated in the special department of the Hospital Sant Orsola of Bologna, and makes the following remarks as regards the local treatment of the skin manifestations: " In soft ulcers, iodoform combined with glycerine was eminently successful (two drachms and a half of iodoform to one ounce of glycerine). Carbolic acid and tincture of iodine were also highly beneficial. Depilation and parasiticidal lotion formed the treatment of the various kinds of favi." The non-syphilitic skin manifestations were attacked according to their fundamental cause: the arthritic by alkalies; the herpetic by arsenical preparations; the scrofulous by anti-scrofulous remedies; with the result of showing the excellence of Dr. Bazin's doctrines.—*The Lancet, April* 12, 1873.

Poisonous Inhalations for Catarrh.—Dr. Channing, in the Boston *Journal of Chemistry*, calls attention to the danger of employing carbolic acid and ammonia in combination in the treatment of catarrh. The agents used separately (by inhalation) are frequently effectual in cutting short an incipient cold. The mixed solutions, however, produce decided narcotic symptoms, which the Doctor explains on the hypothesis that aniline is formed by a reaction familiar to the chemist. A formula for an inhalation consisting of carbolic acid, ammonia, alcohol and water, originating in Berlin, has been quite extensively circulated in the medical journals. Dr. Channing has found carbolic acid alone so effectual that he thinks it hardly worth the while to risk the serious consequences which might follow from the addition of ammonia.

TREATMENT OF WHOOPING-COUGH.—Dr. Berry, of Lancaster, in a letter addressed to the *Medical Times and Gazette*, observes that, in regard to all or most of the remedies employed in the treatment of whooping-cough, the words of Dr. Bateman can be endorsed by practitioners who have had only a moderate experience of this disease. He says: "Perhaps there is no disease for which so many specifics and infallible nostrums are promulgated with confidence, or so few actual remedies known."

Dr. Berry does not pretend to introduce any new remedy for this distressing affection, but desires to speak of one which is simple, safe, and, he thinks, apparently valuable in the treatment of uncomplicated whooping-cough. He has found dilute nitric acid, in doses of from five to fifteen minims, according to age, with simple syrup, given every three or four hours, to alleviate the cough and spasm, and apparently cut short the disease. During a recent epidemic he prescribed this frequently, and has every reason to be satisfied with it. Whether the result has been *propter hoc* or not, he is not prepared to answer, but the cures have certainly been *post hoc*, in the cases in which he employed it; neither does he offer any suggestion as to the *modus operandi* of the remedy, but he believes its action to be that of a tonic, sedative, and antiseptic. He thinks, however, its refrigerating properties should not be lost sight of.

In all the cases Dr. Berry has treated with dilute nitric acid, he has paid attention to the state of the digestive organs, and, in such cases as have required it, he has given an aperient combined with an alterative. There is one advantage in the use of a remedy like this for children: unlike hydrocyanic acid, it is safe, and no unpleasant results need be dreaded from its administration. At the same time it is inexpensive; a consideration in cases in which a large quantity of medicine is likely to be required.—*Medical and Surgical Reporter*.

MODE OF ADMINISTERING CREOSOTE.—As creosote is now frequently employed in the treatment of typhoid fever, and is exceedingly distasteful to some patients, it may be worth while to mention here a formula which in a great measure covers its flavor, and is easily prepared. Creosote, three drops; essence of lemon, two drops; orange-flower water, one ounce; spring water, three ounces. A spoonful to be taken at frequent intervals throughout the day. *Canada Medical Journal.*

EXOPHTHALMIC GOITRE.—J. Forsyth Meigs, M.D., Physician to the Pennsylvania Hospital (*Philadelphia Medical Times*), in his clinical lecture "On a Case of Exophthalmic Goitre," says that, in his later cases, he has used digitalis with benefit. Digitaline granules, gr. 1-60, three times a day, sometimes answer perfectly.

Post-partum Hemorrhage.—Dr. F. B. Walton (*Nashville Medical Journal*) writes: "To guard against this untoward result, I have been in the habit, for more than twenty years, of resorting to the following simple device, viz.: So soon as the placenta is removed and the womb fully contracted, I cause the patient to place her hand upon the uterine globe, and examine its size, density, etc. I then explain to her, in simple language, the cause and nature of flooding—in a word, that it results from relaxation, and consequent enlargement and softening of the organ. I further direct her to keep her hand upon the uterine globe, or at least to make frequent examinations; and in case she commences flooding, or the womb becomes soft and enlarged, to press and rub it until the flooding ceases, or it contracts to its proper size and density; and, if needs be, cause a stream of cold water to be poured from a height upon the naked abdomen. I thus make her a sentinel over her own life, and I have uniformly found her a reliable one—so much so, that, since resorting to this expedient, I have but once been recalled to my recently-delivered patient on account of flooding, and that was a case of internal hemorrhage detected by the patient herself, before any serious consequences resulted.

Fibrin as a Dietetic.—Dr. Goodman, writing to the *British Medical Journal*, says that artificial fibrin is an admirable dietetic substance, being unparalleled for lightness and digestibility, and a great delicacy besides. It is obtained by exposing albuminous material to the action of cold water for a time, the hen's egg, from its great abundance, being the most suitable source of the albumen. When the contents of an egg are immersed in cold water twelve hours or thereabouts, they undergo a chemico-molecular change, becoming solid and insoluble; a change indicated by the opaque and snowy whiteness of the white. The action of heat to the boiling point is now brought into the process, and the fibrin is then ready for use. In cases of deficient nutrition and rejection of food, Dr. Goodman says this artificial fibrin is of the greatest service, as the weakest stomach is able to retain it, and its use appears to promote the appetite for food.

Subacute Pleurisy.—Tonics are regarded as an essential element in the treatment of this affection, (quinine and iron chiefly), and their administration is made the leading feature. The utility of tapping is looked upon as questionable; at all events, it is not to be resorted to early. Diuretics are administered only for the purpose of maintaining the quantity of urine at its normal standard. When a diuretic is required, infusion digitalis is the one commonly employed. Some of the potassa salts are combined with it, if not sufficiently active when administered alone.

Local use of Chlorate of Potassa in Open Carcinoma. This remedy has been used by Dr. Burow, Sen., in five cases of cancerous ulcerations, one twenty-two centimeters in circumference, on the upper arm, three on the mamma, one originating from the periosteum of the upper maxilla and os zygomaticum, which, with the exception of two, had terminated in ulceration without previous operations. It is applied once daily, finely pulverized, over the entire surface of the ulcer. The results of his observations are that the energetic use of chlorate of potassa on these ulcerating cancers produces always diminution and shrinking of the exuberant granulations, resorption of the surrounding infiltrations, lessening of the secretions and the irritability. He does not consider the value of the remedy established beyond a doubt by his yet limited observations, but calls the attention of the profession to his success, in order to insure further investigation.

In a later number of the same journal, Dr. Dorger, of Hamburg, states that French physicians have used the chlorate of potassa ten years ago, according to their statements, with the same effect upon carcinoma as Dr. Burow. The *Union Medical*, 1863, No. 154, contains the results of the observations of Bergeron, Milon and Blondeau, on the treatment of carcinoma by chlorate of potassa. They used very strong solutions of this salt locally, and obtained cures within a few months. Debont's, Lebbane's, Cook's, Charcot's, Delpeche's and Wishon's observations coincide fully with those of Dr. Burow. The conformity of these therapeutical results of so many makes further trials in the interest of the sufferers with this dreadful malady highly desirable.

Treatment of Infantile Cholera.—Dr. Peltier recommends in the above disease a double plan of treatment, consisting simultaneously of tonics and absorbents. He uses the subnitrate of bismuth internally (seven grains to half a drachm in a mixture) and in enemata, beaten up with the yolk of an egg. At the same time he occasionally employs enemata with starch or krameria. The tonics consist of the juice of beef, raw meat (one ounce to an ounce and a half twice daily), and syrup of pepsin (half an ounce to an ounce daily, in teaspoonfuls). He also advocates the simultaneous use of collodion paintings over the abdomen.—*Mouvement Médicale, Dec.* 7, 1872.

Phosphorus Pills.—Dissolve one grain of phosphorus in half a drachm of chloroform; triturate the solution in a mortar with two scruples of powdered liquorice root, until all the chloroform has evaporated; to this add a drachm of powdered soap, and work the whole into a mass with water and divide into twenty-four pills. These pills will keep, are easily made, and evolve no fumes.

Carbolic Acid in Cerebro-Spinal Meningitis.—Dr. C. H. Ladd (*Charleston Medical Journal and Review*) gives the following "*experience*":

"The pertinent facts of these six cases may be thus briefly summarized:

"1st. Of four cases of cerebro-spinal meningitis, two above mentioned and two reported by Dr. Madden, in which the usual remedies were tried, two died within eight days, one made a slow recovery, and one was rapidly growing worse when carbolic acid was substituted for other treatment.

"2d. Of three cases treated with carbolic acid, two from the commencement and one after the usual remedies had proved unaviling, the recoveries were rapid and uninterrupted in each.

"The obvious conclusion is: first, that the usual remedies, as the experience of physicians teaches, seem to have little or no influence in arresting the disease; second, that three of the six cases above mentioned were of a much milder nature than the others; or, third, that carbolic acid, blisters and saline cathartics do, in combination, if not singly, exercise a measurably controlling influence.

"I am disposed to attach much importance to the carbolic acid—to attribute to it a larger influence than to the blisters or cathartics—because, first, in each of the cases in which it was used, blisters were not applied until the third or fourth day, until, in fact, the acuteness of the attack had terminated; and, second, in one, (the first case treated with carbolic acid), the disease was on the increase, although vesication from a blister had existed for two days, amelioration of the symptoms following only, and almost immediately, upon the use of the carbolic acid.

"Blisters and cathartics (I have given the preference so far to sulph. magnesia) have appeared to me to have about equal value in relieving headache and inducing quietude of the nervous symptoms, and will, therefore, should other cases fall under my care, try simply the carbolic acid in $\frac{1}{4}$ to 1 grain doses, and sulph. magnesia in mildly cathartic doses to relieve constipation.

A word as to the diet in the disease, and a suggestion as to its pathology: I ordered as much liquid, nutritious food as the enfeebled condition of the digestive apparatus would allow.

Disinfectant.—Dr. Muscroft, of Cincinnati, Ohio, (*Clinic*), recommends a disinfecting lotion composed of one ounce and a half of the bicarbonate of soda and one ounce of powdered alum, dissolved in water. He uses this mixture for every kind of sloughing sores.

Burns.—The following lotion is used at Charity Hospital, New York: ℞. Sulphate of zinc, grs. xv.; co. spts. lavender, ℥i.; aqua, one pint. M.

Tincture of Iodine.—At the last session of the Academy of Medicine in Paris for the year 1872, Olivade reported several cases of elephantiasis Arabum which he had treated successfully with large doses of the tincture of iodine. He employed it internally and externally. Commencing at first with a few drops, he gradually increased the dose to a drachm, and the success was proportioned to the amount given. The tincture was applied externally on long strips of linen. In every case, marked improvement showed itself by a diminution in the size of the affected part, and by the altered character of the skin. After the lapse of three months, during which moderate compression was kept up, the disease had nearly entirely disappeared.

How to Remove Adhesive Plaster.—Every surgeon, doubtless, is familiar with the appearance of a part which has been enveloped in adhesive plaster, after the straps have been removed. The appearance is not one in very good keeping with a cleanly and neat surgical dressing. The portion of the plaster which is left adhering to the skin may be quickly and completely removed by the use of oil of turpentine and sweet oil. Use a little more than half turpentine. This compound, carefully rubbed over the parts with a bit of cloth or sponge, and then washed off with warm soapsuds, will leave the surface as clean as Nature ever intended.—*New York Medical Record.*

Efficacy of Ice in Gastralgia.—Dr. Joulin proposes a new method of treating gastralgia as follows: 1. A cataplasm of ice morning and evening, ten minutes each time, over the epigastrium. 2. A sinapism immediately after removal of the ice and upon the same surface. The sinapism to be kept on as long as possible. 3. Crushed ice in tablespoonful doses morning and evening, every alternate five minutes for an hour. The crushed ice may be substituted by a water ice flavored to taste. 4. Mustard baths three times a week.

The author claims remarkable results by treatment in this way. *France Medicale, June* 7, 1873.

Liquor Picis Alkalinus.—Dr. L. D. Buckley, of New York, gives the following formula for this preparation, which was originally devised by his father: ℞ Liquid pitch ℥ij; caustic potash ℥j; water f℥v. Mix and dissolve for external use. This mixes with water in all proportions, and ouly moderately discolors the skin. It dries rapidly and leaves very little stickiness. He has used it in all degrees of strength, and regards it as the best preparation of tar.—*Archieves of Sci. and Pract. Med., Feb.* 1873.

Gelsemium.—Dr. E. A. Anderson, Wilmington, North Carolina, esteems this highly valuable in intermittent and other fevers. He begins the treatment of these fevers with twenty-five to thirty drops of the tincture for a grown person, repeated every hour for the first six hours, until one hundred and fifty to one hundred and eighty drops are taken in the day. A child of fifteen years would take about ten drops every hour for six hours; a child of four years about five drops, and an infant one to two drops.

Horehound in Coughs of Children.—Horehound, the herb of *Marrubrium Vulgare*, is an old remedy, and it is doubtful if we have any other that surpasses it as a remedy for the winter and spring coughs children are so much subject to. It has a decided soothing influence on the laryngeal and pneumogastric nerves. A strong decoction of the herb, sweetened with honey, makes an excellent cough remedy which children seldom refuse, and it cures their coughs without much delay.—*Medical Archives.*

Digitalis in the Dropsies and Nephritic Diseases of Children.—Dr. Cooper, of Indianapolis, claims for digitalis a specific action for curing the dropsies and other nephritic complications, arising as a sequel from scarlatina. He gives several cases where ascites, anasarca, albuminuria and emaciation rapidly disappeared immediately after the patient was put on small doses of tinct. of digitalis; without any other treatment the cases recovered perfectly.—*St. Louis Medical Archives.*

Fluid to Restore Putrid Tissues.—Dr. B. W. Richardson gives the following formula for a restorative fluid, which he says will render soft, putrid tissues firm and inoffensive, so that they may be readily dissected: ℞. Iodine, ʒi.; methylated ether, sp. gr. .720, f℥x.; absolute alcohol, f℥x.; strong sulphuric acid, f℥iv. Dissolve the iodine in the ether and the alcohol mixed together, then slowly drop in the sulphuric acid.—*New Remedies.*

Preservative for Milk.—According to A. Hirschberg (*New Remedies*), the addition of fifteen grains of boracic acid to two pounds (equaling a quart) of milk will keep it sweet in hot weather for five days. The usefulness of the milk is said not to be impaired, but the cream rises more slowly than normal.

Linimentum Ammoniæ Iodidi.—℞. Iodine, fifteen grains; alcohol, eight ounces; camphor, two drachms; oil lavender, oil rosemary, each, one drachm; water ammonia, one ounce; mix. This is used largely by many physicians, and is one of the cleanest and most beneficial stimulating liniments.

Camphor Powder in Hospital Gangrene—M. Netter states that he was called in consultation to a case of trauma, in which the patient was attacked with this grave complieation, and in which the surgeon in attendance feared the result, notwithstanding the employment of the ordinary means of perchloride of iron, carbolic acid, &c. The appearance of the wound called to mind the phagedænic syphilitic ulcer. But in this last form a particular remedy succeeds admirably, namely, the free application of powdered camphor, that he has hitherto employed empirically and against this affection alone. This was tried in the case just mentioned, and with perfect success. A second case attended by fortunate results has been recorded by a well-known naturalist, M. le Vaillant, who had care of the wounded in the Hospital of St. Mals. In a third case, which proved equally successful, M. Netter noted a peculiarity which may perhaps afford an explanation of the mode in which camphor effects a cure. The dry matter of hospital gangrene liquefies in contact with camphor, in virtue, no doubt, of the well-known action of camphor on the fats. Hence camphorated ointments must be kept in dark places, whilst pure lard is unaffected by exposure to ordinary conditions. The camphor may also, perhaps, destroy the peculiar ferment. Before applying the camphor to wounds affected with hospital gangrene, they should be lightly syringed with water.—*Comptes rendus, Practitioner.*

Muriate of Iron in Erysipelas.—Dr. T. J. Stevens (*Medical and Surgical Reporter*) writes: I think this medicine is the nearest a specific of any I have found in an experience of forty years. I have been very successful in this complaint by treating it as follows: I begin with a dose of twelve drops, which I increase to thirteen in two hours, and so on, increasing one drop every two hours, night and day, until the disease stops spreading. I have never yet exceeded forty-five drops at one dose. After the disease has run four days, I either discontinue the iron and give quinine, or give quinine simultaneously with the iron. With regard to external treatment, I have not much faith in the efficacy of any kind, but in some cases I have used to good advantage the sulphite of soda, half an ounce to a pint of water, applied as a wash. The average length of time of sickness by this treatment has been about six days, the extremes being four to eight days.

Simple Cerate.—A modification of this dressing is used which renders it much more agreeable, and much easier of manipulation in cold weather. It consists in the addition of oil of almonds in the proportion of ʒi. to ℥i. This makes one of those little variations in surgical dressings which may add to the comfort of the patient and the convenience of surgeon.

Old French Remedy for Anasarca.—Take three grains of hermes mineral (French, American will not do) in three tablespoonfuls of honey and syrup. Give one teaspoonful an hour before breakfast, one an hour before dinner, and one an hour before supper. Should this procure three or four operations from the bowels, the quantity will be sufficient; otherwise increase the quantity. The efficiency of the medicine consists in giving the patient a sufficient quantity to purge him three or four times in the twenty-four hours. Should the hermes excite the circulation and render the pulse full, a decoction of the following is substituted until the febrile symptoms subside: ℞. Yellow-dock root, (dried) two ounces; water, two quarts; reduce by boiling to one, and give two to three ounces, more or less, three times a day, to produce three or four operations a day. When the fever subsides, return to the hermes. The diet should consist of dry light-bread (no salt or butter) with broiled bird, chicken, etc., without butter or lard; half a cup of strong coffee three times a day. No water is to be used. As much exercise as possible short of fatigue.

Theory and Cure of Diabetes Mellitus.—The theory and cure of diabetes, *Progresso Medico* of July), proposed by Professor Cantani, has met with great favor. He says: 1. In diabetes mellitus there takes place the formation of a special kind of glucose which might be called paraglucose. 2. All diabetes mellitus essentially is due to a diet containing amylaceous and sugary matters. 3. Every diabetic organism which is forced, by means of an excessive meat diet, no longer to form paraglucose, ought finally to recover.

Cantani is profoundly convinced of the idea of the formation of paraglucose. He believes that in healthy individuals there is true glucose in the blood, but in diabetes there is a false glucose, which is but little oxidable, and passes out of the system just as quinine, iodide of potassium, etc., does.

Dr. Primavera contests the idea that we daily introduce much glucose into the organism, even when we eat much starch or sugar. What goes into the blood is not true glucose, says he, except in very small quantities. All the rest which enters by the vena portæ and through the liver is there transformed in two ways. It must, therefore, be looked on as a kind of peptone. He remarks that in every diabetic patient there are two stagee, the first always ceases when amylaceous or sugary foods are suppressed, and in the second the cessation of such diet does not entirely cause a stoppage of the diabetes mellitus.

Dr. Balfour, of Edenburgh, relates cases of complete cure from the use of Cantanti's method (a strictly animal diet, where from one to two pounds of meat a day are used, and from one to four spoonsful of lactic acid are given to drink in a cup of water occasionally.

